# Supplementary material for: Polariton condensation and surface enhanced Raman in spherical ZnO microcrystals
Source: Nat Commun. 2020 Sep 30;11:4908. doi: 10.1038/s41467-020-18666-4 (PMC7528097; doi:10.1038/s41467-020-18666-4)
Supplement: Supplementary file 1 — Supplementary Information [file 41467_2020_18666_MOESM1_ESM.pdf]

Supplementary Information

Polariton condensation and surface enhanced Raman in spherical ZnO microcrystals.

Volkov, Oliver and Perry

Supplementary Note 1. SYNTHESIS OF MICROCRYSTALS AND DODECAPEPTIDE

The ZnO polycrystalline microspheres were fabricated based on a protocol from the literature [1]. A reaction mixture (10ml) containing 50 mM $Zn(NO_3)_2$, 50 mM HMTA and 38 mM sodium citrate was prepared and mixed at room temperature (22 ^oC). The solution was heated to 90^oC for 1.5 hrs and the precipitate formed collected by centrifugation using Centrifuge 5904R M (Eppendorf, Hamburg, Germany) at 5000 rpm for 5 minutes. The pellet was washed with 2x 10ml Type-I water, 1x 10ml absolute EtOH before being suspended in 500µL Type-I water and lyophilised. The properties and responses of ZnO microspheres demonstrate great sensitivity to temperature, environment and post processing surface conditioning.

Synthesis of GLHVMHKVAPPR (G12) dodecapeptide was via Fmoc chemistry using micro-wave assisted solid phase peptide synthesis (Liberty1 instrument, CEM Corporation) as reported previously [2].

We conduct confocal emission microscopy using a Leica TCS SP5 station (Leica, Milton Keynes, UK) equipped with a 63x objective (numerical aperture, 0.5). Under the rate of 400 MHz, we scan spatial regions of 124x124 micron with resolution of 0.242 micron in both directions of the image plane. For excitation we use spectrally narrow not-polarized laser radiation at 405 nm. Application of the confocal geometry means that the emission is collected strictly from the area (with diameter of about 200-300 nm) at the excitation point. To explore and understand the nature of the response we detect, in Supplementary Figure 1 we show graphically the experimental geometry. Specifically, in the image we present the case when a signal is stimulated and sampled at the edge of a microsphere while the image plane crosses the centre of the microsphere. The latter is consistent with our experimental conditions: in the article we discuss images sampled at equatorial planes of the microstructures. Since in experiment we employ a non-polarised excitation field, then, at the focus we may describe polarisation of the exciting field with two orthogonal polarisation components, the axes of which would lie in the image plane (which contains the equatorial cross-section). Orientation of the two polarisation axes is arbitrary. However, for practical convenience we should account the spherical symmetry of the system – therefore, we select one of the polarization axes to be parallel to the line from the centre to the focus, and another to be tangential in respect to the surface line. The two blue arrows at the focus cite as shown in Supplementary Figure 1 describe the selected orientation of the

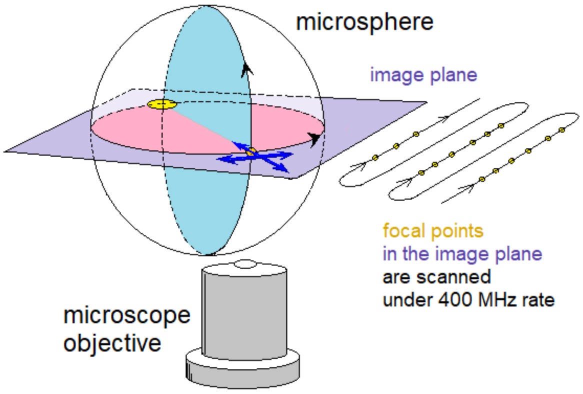

Supplementary Figure 1. **Geometry of confocal emission microscopy experiment.**

polarisation axes of the exciting field. In the numerical simulations (see the following sections) we select the X-axis of the simulation box to be parallel with the polarisation component of the exciting field which is along the radial line; and the Y-axis of the simulation box to be parallel with the polarisation component of the exciting field which is along the tangential line in respect to the curvature of the surface. When we focus the excitation field at the edge of a spherical microstructure we stimulate various responses in matter. According to the described system of coordinates, possible responses can be factored and described in respect to the XZ, YZ and XY planes: the cyan and the pink cross-sections in the Supplementary Figure 1 are co-aligned with the XZ and the XY planes as we will use in the numerical simulations (see further).

Supplementary Note 2. CONFOCAL EMISSION MICROSCOPY IN DEPENDENCE ON EXCITATION WAVELENGTH: BAND-GAP AND SURFACE STATES IN LARGE MICROCRYSTALS

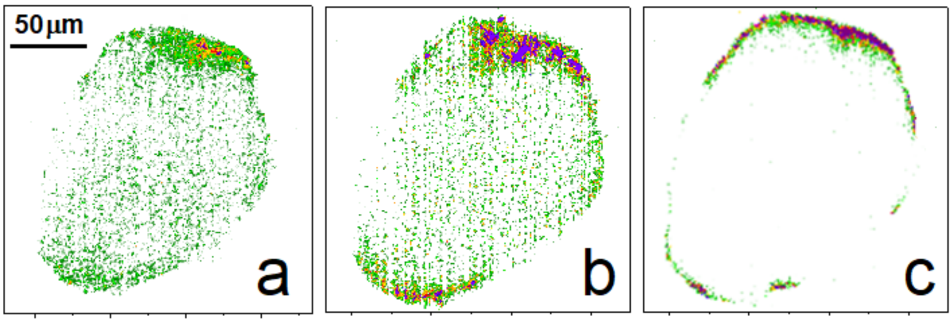

Supplementary Figure 2. **Localizing tendencies in ZnO microcrystals in dependence on excitation and detection wavelengths. a,** Confocal microscopy images scanned at the equatorial plane of the microcrystal detecting at 430 using 405 nm for excitation. **b,** Confocal microscopy images scanned at the equatorial plane of the microcrystal detecting at 510 nm using 405 nm for excitation. **c,** confocal microscopy images scanned at the equatorial plane of the microcrystal detecting at 510 using 488 nm for excitation.

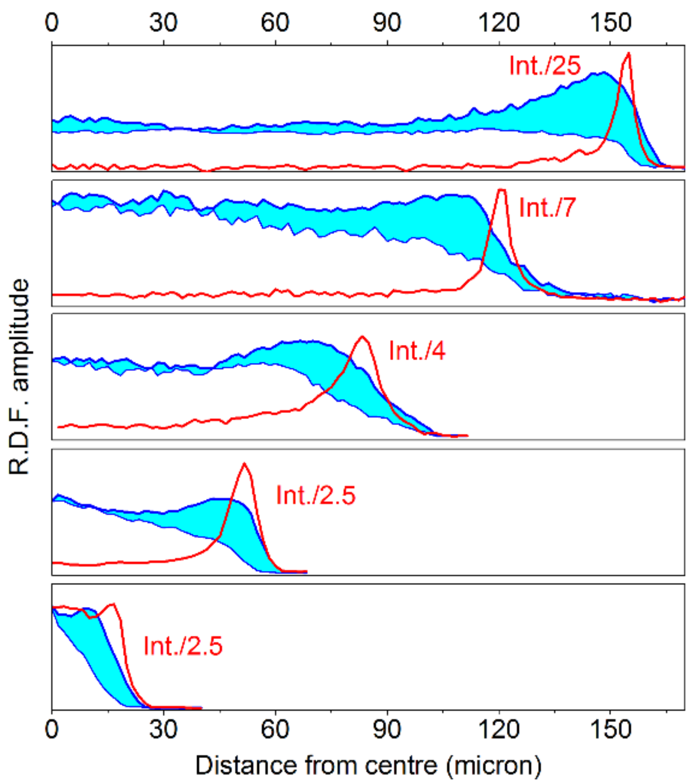

Supplementary Figure 3. **Emission properties in dependence on radius.** Radial distribution function for emission intensity detected at 430 nm under 405 nm excitation (blue lines), and detected at 510 nm under 488 nm excitation (red lines) from Sigma-Aldrich ZnO microcrystals in dependence on size, as shown. Upper and lower blue lines confine cyan filled regions within which emission intensity may vary. The data was taken using intermediate 50% ($2.9 \cdot 10^5$W/cm^2) of laser power.

Supplementary Note 3. NUMERICAL SIMULATIONS

To discuss experimental results we conduct numerical simulations of possible field distributions in micro-spherical structures under different excitation regimes solving finite difference time-domain integro-differential equations as implemented in Lumerical package (Release: 2020a r4, Version: 8.23.2236), Lumerical Inc., suite 1700-1095 W. Pender St., Vancouver, BC, Canada V6E 2M6. Calculations in complete spheres are necessary to understand the relative contributions of different (along different directions) whispering gallery (WG) modes and spherical harmonic (SH) modes in dependence on excitation and sampling conditions and in dependence on radius. We conduct calculations of field distributions in ZnO microspheres (included into the simulation box completely) using Gaussian beams focused at the side, at the bottom and in the centre of microspheres: see Supplementary Figures 4-6. To model field distributions under excitation with a non-polarised field we use two Gaussian beams under orthogonal polarisations placed in the same manner and co-propagated the same. Two blue arrows indicate polarizations of the two Gaussian sources. We describe the orientation of the polarisation axes as shown with blue arrows in Supplementary Figure

1. The magenta arrow indicates the direction of propagation of the Gaussian beams. White circles in the XY, XZ and YZ planes indicate the positions of frequency sampling points. We place the sampling points in the orthogonal planes in an attempt to stress differences in the character of field dynamics

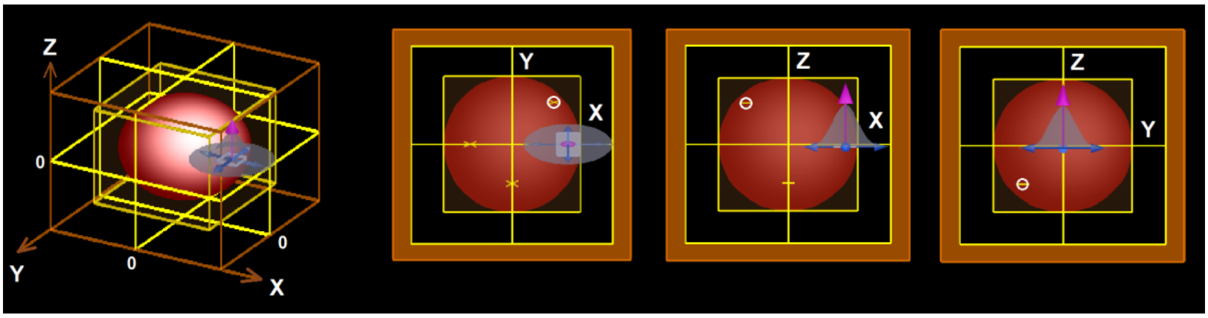

Supplementary Figure 4. **Simulation outline.** Coordinate system, position of a Gaussian beam and planes where fields are sampled.

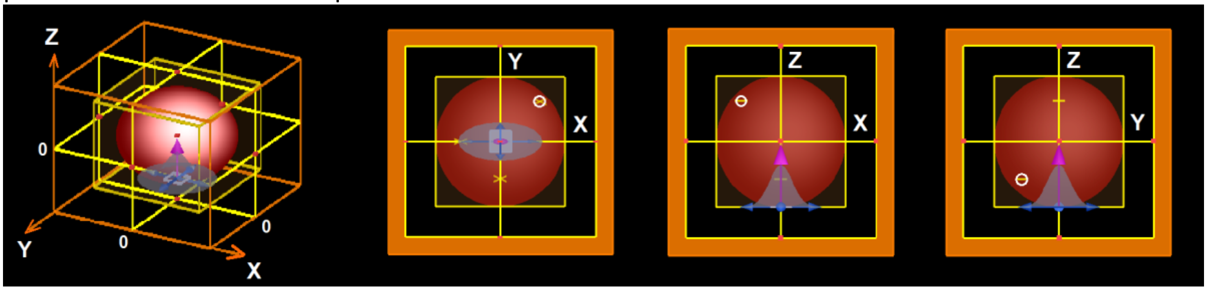

Supplementary Figure 5. **Simulation outline when exciting at the bottom of a microsphere.**

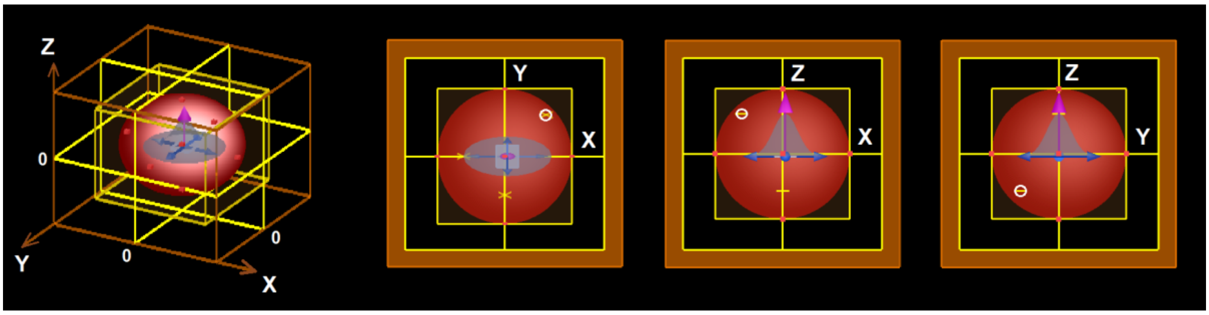

Supplementary Figure 6. **Simulation outline when excitation in the centre of a microsphere.**

To account for the properties of ZnO we adopted refractive indices of refraction in the spectral range from 300 nm to 900 nm, as reported earlier [3]:

https://refractiveindex.info/?shelf=main&book=ZnO&page=Stelling

The indices were imported into Lumerical and fitted to the 12th order with a tolerance of 0.005. In all simulations the boundary of the box was set to a perfectly matched layer condition. Simulations of the field were conducted while the maximum mesh step (along x, y and z directions) was set to 10 nm. To avoid the impulsive part in frequency sampling we adopted time apodization for the start with the centre and a width of 80 and 30 femtosecond respectively. The spatial spans of Gaussian sources (in the basal plane) were set to 400 nm in both directions according to the central wavelength of the

excitation light we used in experiments. The wavelength span for the Gaussian source was in the spectral region between 0.39 and 900 nm.

Supplementary Note 4. NATURE OF BAND-GAP MODE EXCITED AT THE SIDE OF A MICROSPHERE

Let us consider the results of simulations for a microsphere with radius 1.22 micron, as we explore experimentally. Here, we will describe simulation results when we adopt side excitation of its equatorial plane as described in Supplementary Figure 4 under the geometry as shown in Supplementary Figure 1. In Supplementary Figure 7 we show spectra of the modes detected at sampling points positioned in the orthogonal planes as described. One can clearly see that the spectrum in the YZ plane is more is dominated by the lower energy subset of resonances at about 750 nm, while the spectra sampled in the XZ and XY planes have more obvious contributions of the higher energy subset of resonances at about 490 m. We discuss the nature of the cavity modes in dependence on their spectral region in the following section. Here, since in our experiment we reported emission nonlinearities at about 410 nm, we will focus mainly on the description of the cavity modes expressed at 400-405 nm.

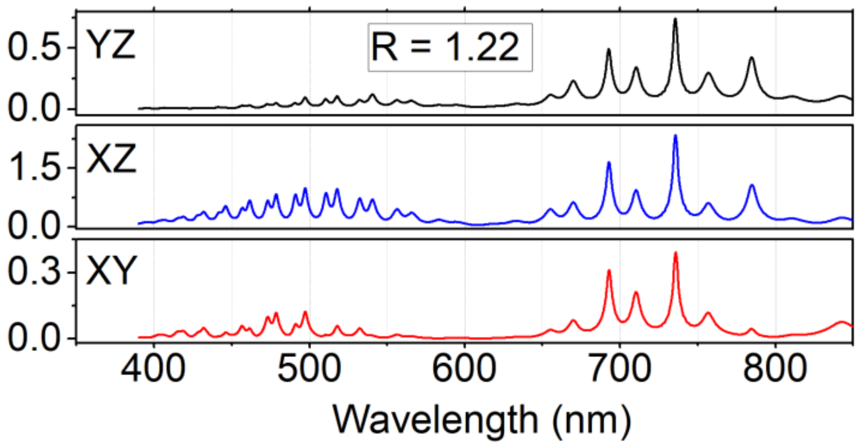

Supplementary Figure 7. **Spectral properties of cavity modes.** Spectra of the modes anticipated at the sampling points in the YZ, XZ and XY planes (see white circle marks in the corresponding panels in Supplementary Figure 4) for a microsphere of R = 1.22 micron when excited at its side under the geometry described in Supplementary Figures 1 and 2.

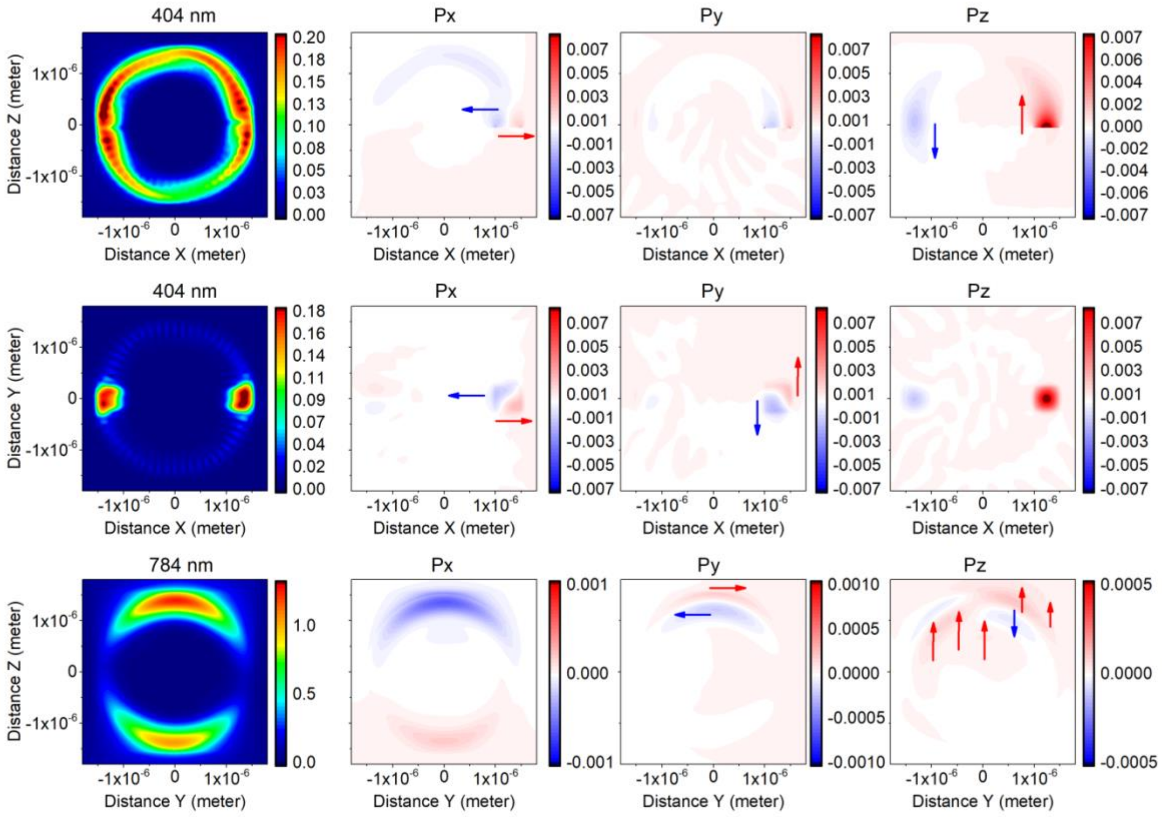

Supplementary Figure 8. **Theoretical studies of energy flux directions.** Images of the cavity modes sampled at the wavelengths as indicated and components (X, Y and Z) of Poynting vectors in the XZ (upper row), XY (middle row) and YZ (lower row) planes calculated for a ZnO microsphere with R = 1.22 micron when excited at its side (see Supplementary Figure 4) under geometry as shown in Supplementary Figure 1. Blue and red arrows are the eye guides helping to indicate physical directions of the indicated components of the Poynting vector in the spatial regions where they take negative (blue) and positive (red) values.

In particular, the left panel in the upper row in Supplementary Figure 8 presents the spatial distribution of the cavity modes at 404 nm. Theory predicts intense field distribution with a periodic pattern along the curved boundary, which is specific to a whispering gallery. The obviously non-uniform (not very circular) character suggests that several modes compete for expression in the indicated spectral region. Examination of simulated pattern suggests dominance of the modes with angular order of about m = 26 ± 2 and radial order n = 1-2. The discontinuity of the intensity at Z = 0 indicates where the exciting field of the Gaussian beams is injected. According to our experimental geometry (see Supplementary Figure 1) the described modes would contribute into the responses we detect only in the image plane where (Z = 0 ± 200 nm): see the small and large yellow spots in Supplementary Figure 1. The high efficiency to stimulate WG modes in the XZ plane is in agreement with the predicted large positive value of the Pz component of the Poynting vector: red spot in the region of field injection in the right image in the middle row in Supplementary Figure 8 indicates that the Z component of the Poynting vector is pointing towards the reader, from the image plane.

Next, the left panel in the middle row in Supplementary Figure 8 presents image specific to the cavity modes at 404 nm anticipated in the XY plane. The image demonstrates two bright spots due to the contributions of the WG modes which propagate in the XZ plane as we have described in the previous paragraph. In the rest of space, the image shows a relatively weak but rather uniform WG cavity mode with angular order m = 56 and radial order n = 1. Careful examination of the simulated pattern suggests also a residual presence of a mode m = 54, n =1. Non-zero efficiency to stimulate WG modes in the XY planes is only due to the curvature within the waist of the Gaussian beam. This is reflected in non-zero Px and Py components of the Poynting vector of cavity modes stimulated under excitation of the Gaussian beam (of two polarisations) propagating along the Z direction.

Finally, here we would like to consider the cavity modes specific to the YZ plane. Considering the spectral properties shown in Supplementary Figure 7, in the left panel in the lower row in Supplementary Figure 8 we present the spatial distribution of cavity modes at 784 nm (we present images for other resonances in a later section). The image clearly indicates that the described mode has the character of a spherical harmonic. Components of the Poynting vector (as shown in the upper row) indicate that under the considered geometry of the experiment one should expect relatively high efficiency in stimulation of such mode: the main part of the Pz component (red fields in the upper part dominate) coincide with the direction of the excitation field, which is behind the YZ plane. According to our experimental geometry (see Supplementary Figure 1), since we detect in the YX plane under confocal discrimination, such a mode will not contribute into the responses we detect.

Supplementary Note 5. SPECTRAL PROPERTIES OF THE COMPUTED MODES

In the previous section we describe three cavity modes (at selected wavelengths) specific to the XZ, XY and YZ sampling planes when the microsphere is excited at its side under the geometry described in Supplementary Figures 1 and 4. In order to reach a broader perspective on the nature of the cavity modes, here we would like to compare spectral and spatial properties sampled at different wavelengths when a Gaussian beam is focused at the side, in the centre, and at the bottom (Supplementary Figures 4-6). The first and second excitation regimes are relevant to our experimental studies, while the simulations when we excite at the bottom are didactic. In the case of excitation at the centre, we provide a small (50 nm) negative placement of the Gaussian beams to remove excessive degeneracy.

First, in Supplementary Figure 9 we compare spectra of cavity modes sampled in the YZ, XZ and XY planes for a ZnO microsphere of R = 1.22 micron.

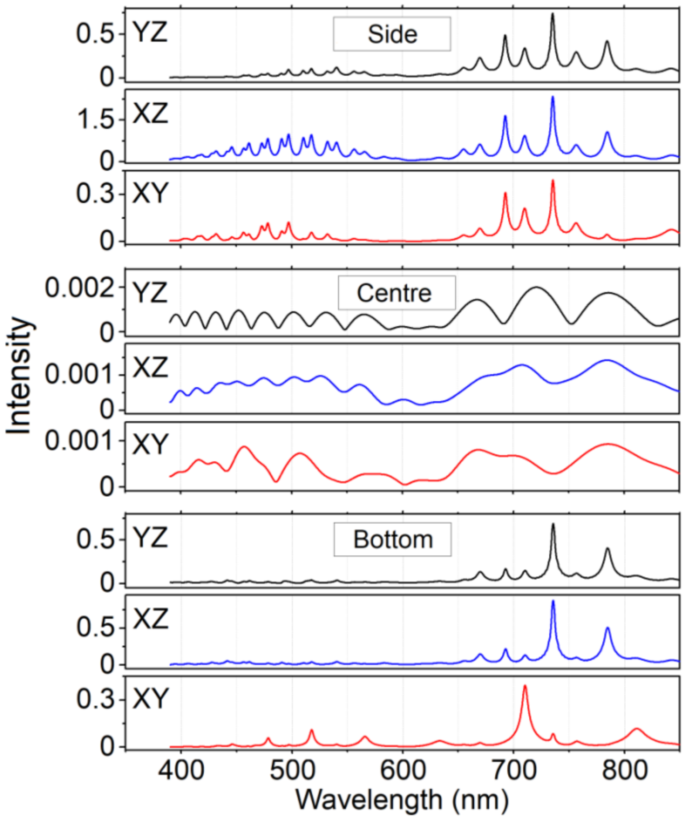

Supplementary Figure 9. **Spectral properties of cavity modes.** Spectra of cavity modes anticipated at the sampling points in the YZ, XZ and XY planes (see white circle marks in the corresponding panels in Supplementary Figures 4-6) for a microsphere of R = 1.22 micron when excited at the side, in the centre and at the bottom: see Supplementary Figures 4-6. The top set of spectra (specific to the YZ, XZ and ZY planes) is the same as in Supplementary Figure 7.

Supplementary Note 6. FIELD DISTRIBUTIONS IN DEPENDENCE ON EXCITATION POSITION

In Supplementary Figures 10, 11 and 12 we image cavity modes at selected wavelengths (as indicated) in the corresponding planes for the three excitation conditions. The simulated spectra suggest that when the system is excited in the centre, the intensity of responses is three orders less intense comparing to such when the system is excited at the side: see middle and upper sets in Supplementary Figure 9. Consistently, images in Supplementary Figure 11 are weaker than in Supplementary Figure 10. It is important to note, that when excited in the centre, the micro-spherical cavity supports modes which demonstrate character of spherical harmonics (SH). At the same time, under excitation in the centre, simulations suggest some localized tendencies. Overall, the simulated results for this excitation

regime are consistent with what we observe in experiment – negligibly small emission in the central region of images taken at the equatorial plane of ZnO microspheres.

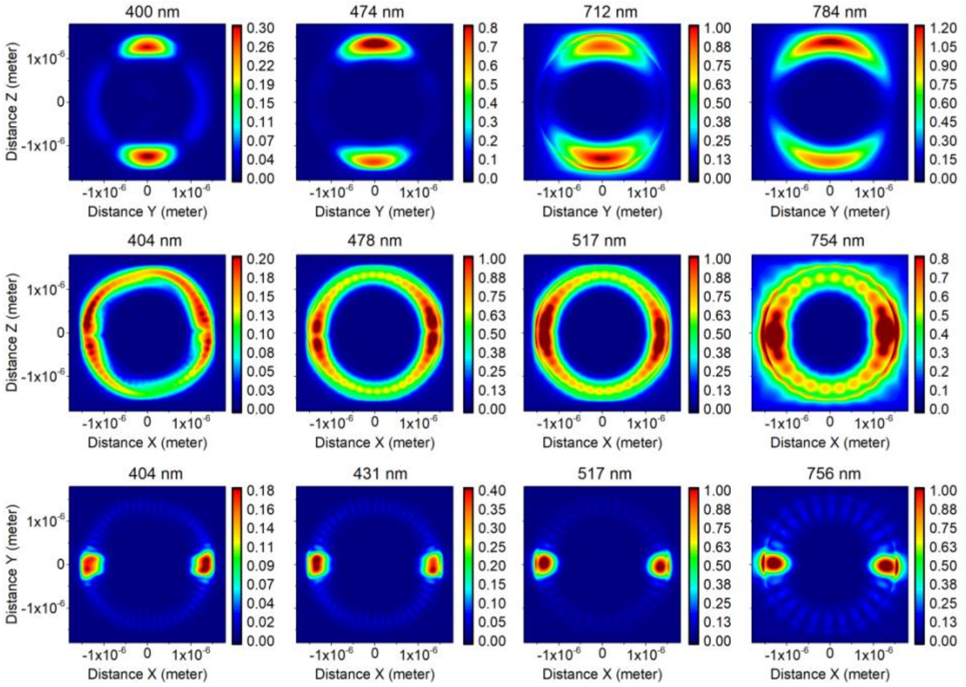

Supplementary Figure 10. **Calculated images of selected modes.** Cavity modes specific to the YZ (top), XZ (middle) and XY (bottom) planes and sampled at different frequencies (see Supplementary Figure 7) when excited at the side of microsphere (see Supplementary Figure 4) of 1.22 micron under the geometry described in Supplementary Figure 1.

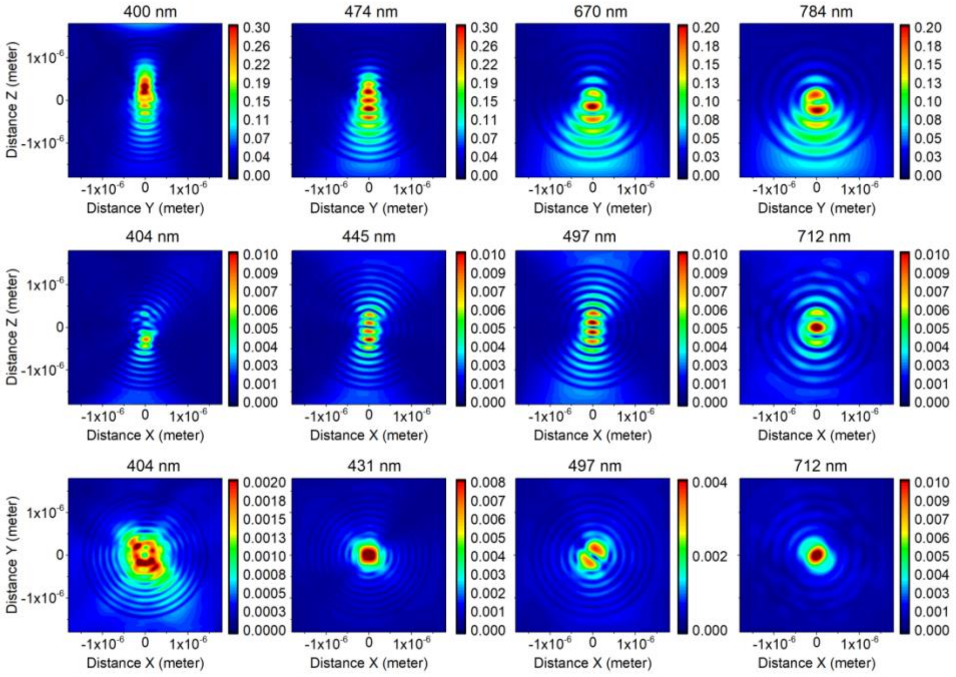

Supplementary Figure 11. **Calculated images of selected modes.** Cavity modes specific to the YZ (top), XZ (middle) and XY (bottom) planes and sampled at different frequencies (see Supplementary Figure 9) when excited in the centre of a microsphere (see Supplementary Figure 6) of 1.22 micron under the geometry described in Supplementary Figure 1.

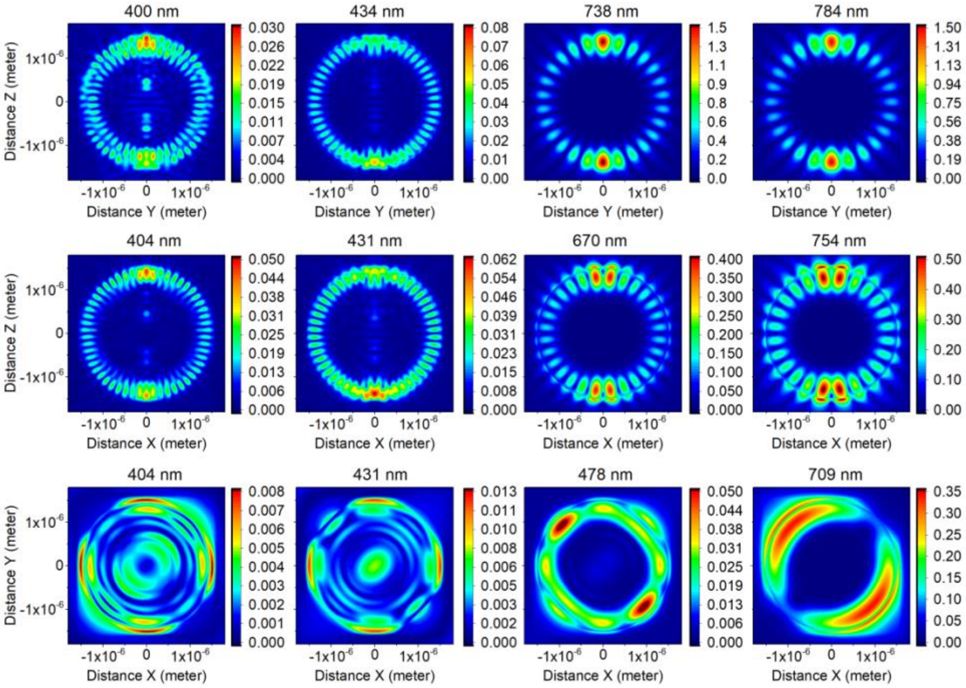

Supplementary Figure 12. **Calculated images of selected modes.** Cavity modes specific to the YZ, XZ and XY planes and sampled at different frequencies (see Supplementary Figure 9) when excited at the bottom of microsphere (see Supplementary Figure 5) of R = 0.6 micron (left) and 1.22 micron (right) under the geometry described in Supplementary Figure 1.

Considering spectral dispersions (Supplementary Figure 9) and spatial distributions (for example, see Supplementary Figures 10 and 11) it is clear that WG and SH modes compete for expression in the whole spectral range: theory predicts that each type may find expressions in the ultraviolet and in the visible spectral ranges. However, apparently, SH modes have stronger intensities in the visible.

Theory predicts that spherical harmonics would overwhelm in the XY plane when excitation is focused at the bottom according to geometry shown in Supplementary Figures 1 and 5. As we have noticed before, in our experiment we did not employ such an excitation regime. Consistently, the experimentally scanned images do not show any strong emission as predicted SH modes at 404 nm in the XY plane would demonstrate (Supplementary Figure 12).

As a result of the simulation studies we can conclude here that in our experiment on microspheres of about 1 micron radius we should expect a strong emissive component due to WG cavity modes whenever the beam focus would approach the edges of a microsphere. Such strong emissive components would be a signature of the place where the WG modes (developed along the corresponding XZ plane) would cross the imaging (XY) plane: for example, see the bright spots in the lower row of images in Supplementary Figure 10. At the same time, due to the curvature and due to the coupling between WG modes in the XZ and XY planes at the cross-points (notice that the bright

spots show a spatial substructure which is according to the lobes of the WG modes in the XY plane), in the image plane we also detect a weaker but present contribution of the WG modes specific to the XY plane. The contributions of such modes is smaller and according to differences in intensities of the spectral peaks as shown in the UV region for the spectra detected in the XZ and XY plane: see the upper set in Supplementary Figure 9. The described contributions of the cavity modes would dominate emission at edges - the rim image pattern as detected in experiments on microspheres.

Supplementary Note 7. COMPARISON WITH THE EXPERIMENTAL RESULTS

In experiment we scan the image plane line by line with resolution 242 nm as we described in the previous section. To model how the anticipated cavity modes in ZnO microspheres would contribute into a detected image considering just one scanning line (for convenience we take it to pass through the centre of the structure, in Supplementary Figure 13 (at the left) we describe a simulation where we place an array of Gaussian sources from the centre toward the edge along the X axis every 242 nm. In the middle panel in Supplementary Figure 13 we present a complete image generated by the array of Gaussian sources: since under confocal setting, the microscope would collect responses along the orange line, starting from the centre toward the right edge, every 242 nm where we place the Gaussian sources. In the right panel in Supplementary Figure 13 we compare high resolution FDTD data along the orange line (top), average projection of high resolution FDTD data onto the experimental grid (middle) and corresponding data from the experiment (bottom).

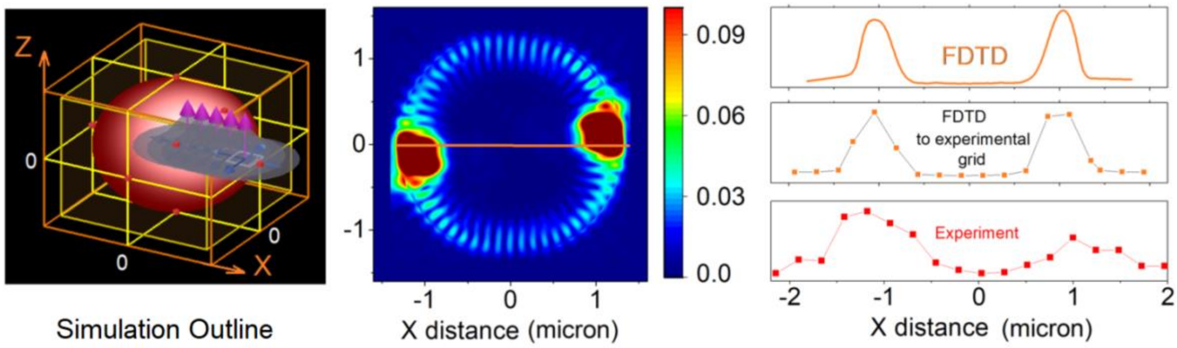

Supplementary Figure 13. **Comparison of theory and experiment.** Left, simulation setting. Middle, computed complete image and indication where the selected reading line (orange line) is passing. Right, comparison of theory with experiment.

Comparison of FDTD theory prediction with the experiment shows a reasonable agreement, even though the simulated features are narrower. Here it is necessary to account that the simulation is only for the field component, while the experiment reports on contributions of fields and of quasi-particles (excitons). Such contributions may be coupled (polariton) or not. In the latter case, contributions of

quasi-particles would be according to their physical motion and energetic relaxation. Additionally, since in confocal microscopy experiments we sample a certain range along the Z axis, the broadening towards the centre may indicate contributions of excited states and field components which are next, above and below, the equatorial plane.

Supplementary Note 8. DEPENDENCE ON RADIUS: FIELD CONFINEMENT

Here we explore the effect of size (radius) on the spectral-spatial properties of the cavity mode in ZnO microspheres. First in Supplementary Figure 14 we present spectral expressions of the modes stimulated in the XY plane under the side excitation.

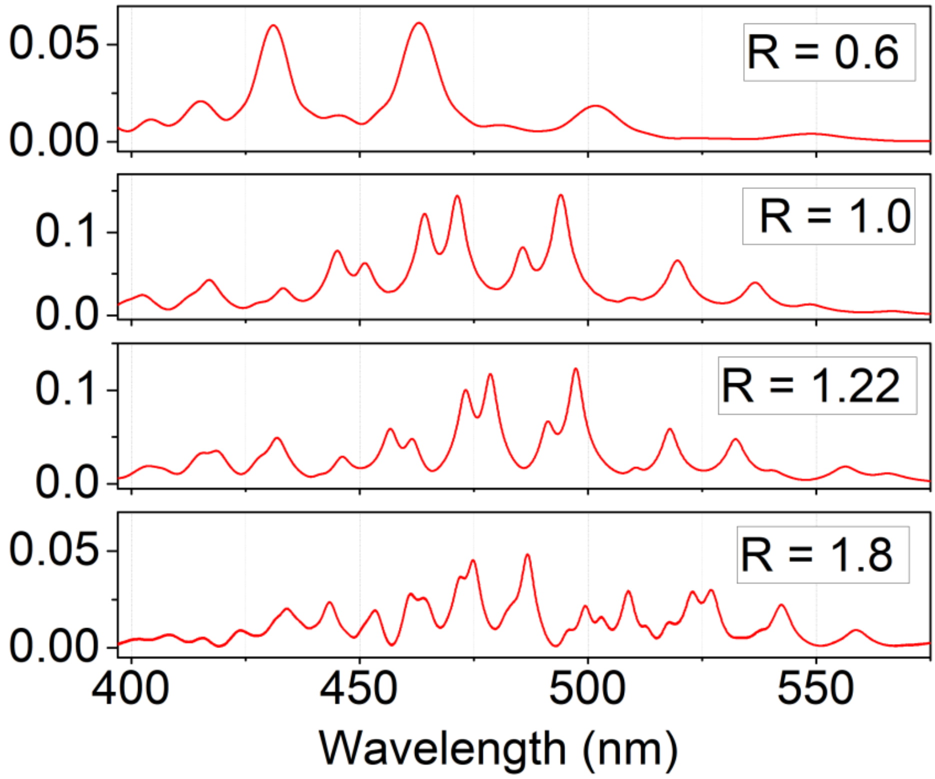

Supplementary Figure 14. **Spectral properties in dependence on radius.** Spectra of the cavity modes in UV-VIS spectral range detected in XY plane in dependence on radius.

According to FDTD theory, a micro-spherical cavity of a smaller radius would support a smaller number of modes which would tend to have higher intensity at the lower wavelength side, and would tend to become spectrally broader.

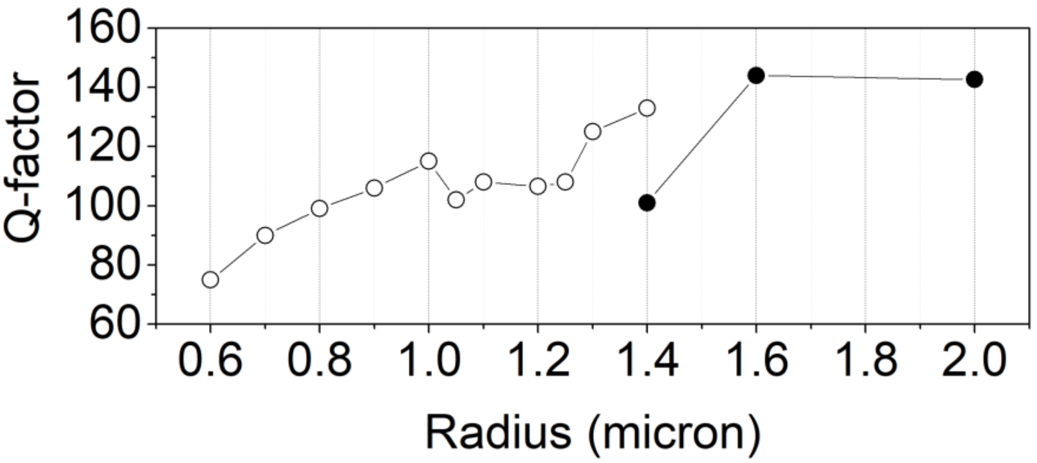

Supplementary Figure 15. **Quality factor in dependence on radius.** Open circles present Q-factors calculated for the cavity modes expressed in the UV spectral range (between 404 and 450 nm) calculated in in the XZ plane in dependence on radius of a complete microsphere. The quality factors for the modes in the XY planes are of comparable values. Closed circles are Q-factors calculated for the cavity modes expressed in the UV spectral range (between 404 and 450 nm) calculated in a layer of 800 nm thick selected in the XY equatorial plane of a microsphere in dependence on the radius of the microsphere.

In Supplementary Figure 15 we present computed quality (Q) factors for the cavity mode stimulated in the UV spectral range in the XZ plane (values for the XY plane are about the same) under the side excitation. Evaluation of Q factors was according to the method reported by V. Mandelshtam and H. Taylor [4]. The computed values are in the range of those reported for high quality cavities designed to demonstrate polariton condensation regime [5]. In our studies, however, we take advantage of the spherical boundary [6,7] of chemically prepared ZnO microstructures to support the cavity modes effectively.

Supplementary Note 9 FOURIER TRANSFORM ASSISTED ANALYSIS ON SPATIAL PROPERTIES OF EMISSION IN MICROCRYSTALS ON POWER.

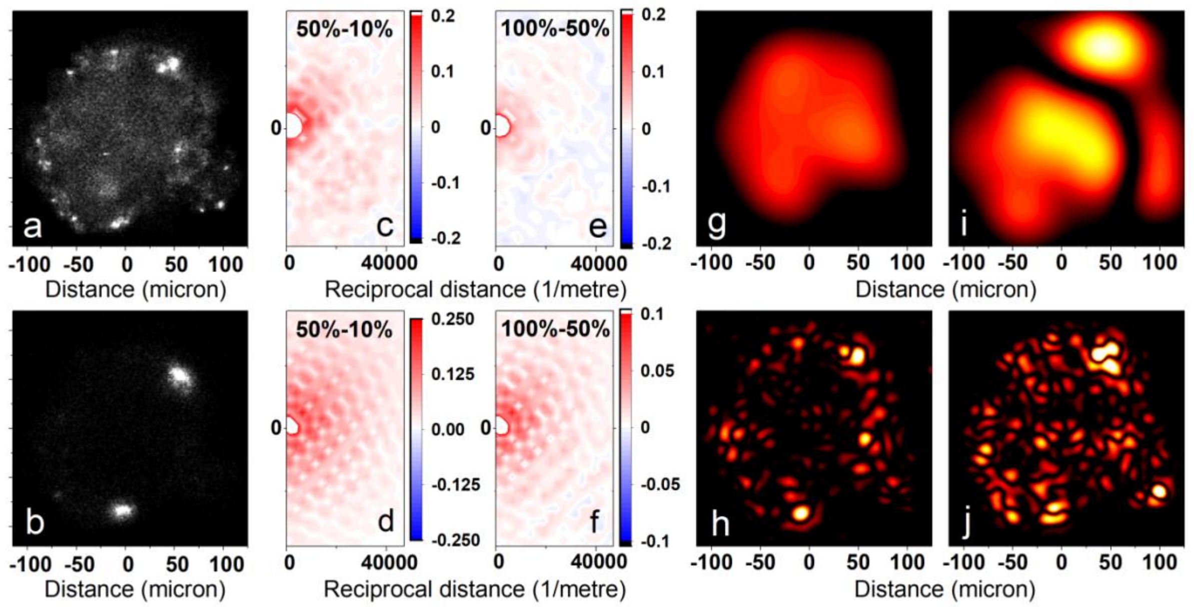

Supplementary Figure 16. **Fourier transform factored power-dependent redistribution of emission spatial components in a ZnO microcrystal. a** and **b,** Confocal emission scans for the ZnO microcrystal detected at 430 and 530 nm, respectively, using 405 nm for excitation. **c** and **d,** Differences of normalized 2D spatial frequency spectra (by Fourier Transform of images) of images taken in the ZnO microcrystal using 50% ($2.9{\cdot}10^5$W/cm^2) and 10% ($5.8{\cdot}10^4$ W/cm^2) of laser power. **e** and **f,** Analogous differences of normalized 2D spatial frequency spectra while using 100% ($5.8{\cdot}10^5$ W/cm^2) and 50% ($2.9{\cdot}10^5$ W/cm^2) of laser power. Normalisations were conducted taking sums of intensities of all pixels. A 2D spatial spectrum is a radial function: here, we position zero frequency in the middle of the left vertical axis. **g** and **h,** Inverse FT after applications of the low frequency path filter (from 0 to 1.2 10^4 m^{-1}) to the difference of emission images detected at 430 nm upon excitation power increase from 50 to 100%, and from 10 to 50%, respectively. **i** and **j,** Inverse FT after applications of the band-pass filter (from 10^4 to 5.2 10^4 micron^{-1}) to the difference of emission images detected at 430 nm upon excitation power increase from 50 to 100%, and from 10 to 50%, respectively.

Supplementary Note 10. MOMENTUM SPACE REPRESENTATION ON RADIUS.

In Supplementary Figures 17 and 18 we provide a systematic account for images of the cavity modes stimulated under the edge excitation in the UV-VIS spectral region (at the wavelengths as indicated) and their momentum space spectra in dependence on radius as sampled in the XZ and XY planes, respectively. Momentum space spectra indicate that a mode of the same frequency but in a smaller microsphere would be spatially broader along the radial dimension. Practically, this means that they would need a larger relative fraction of volume to occupy.

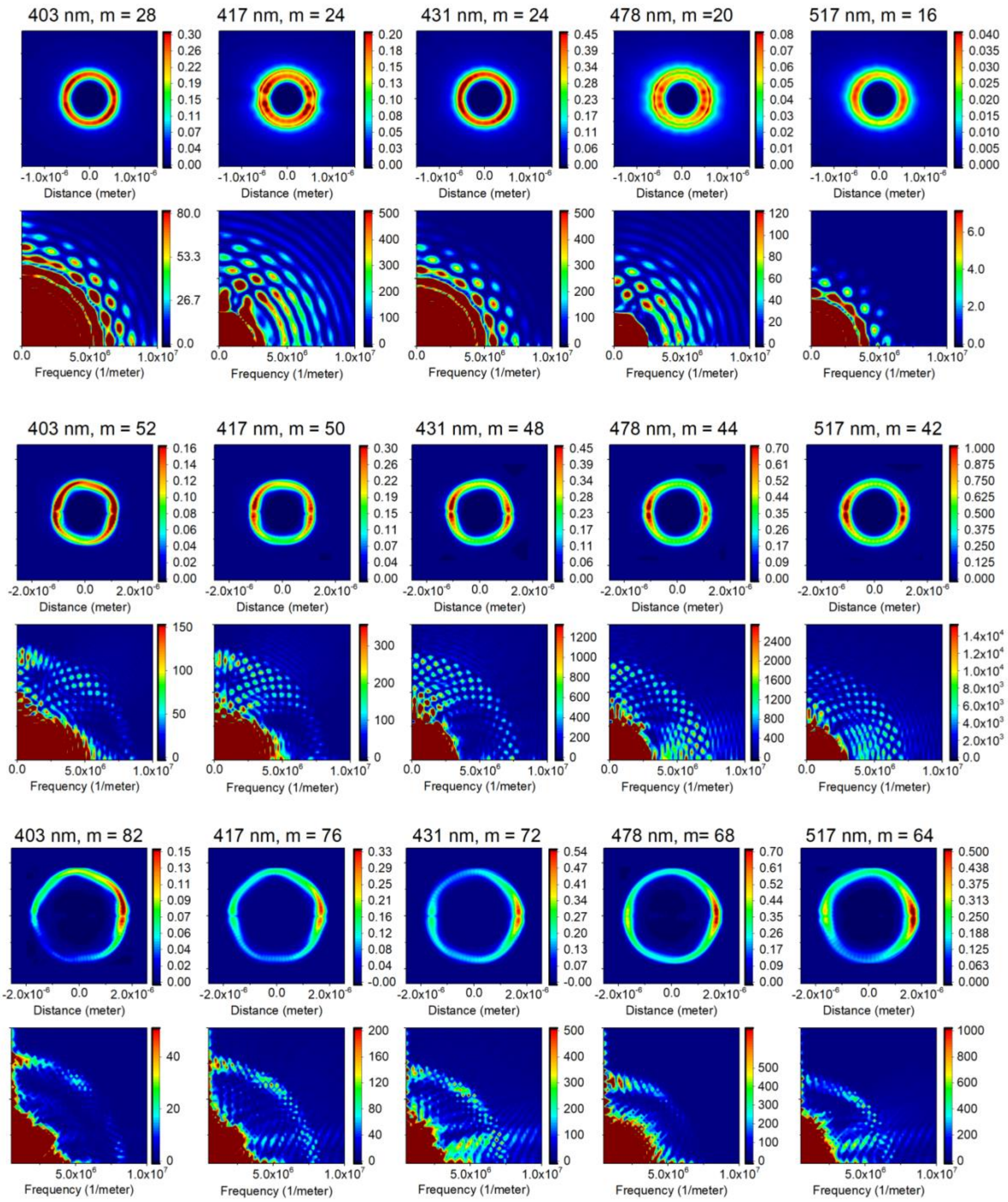

Supplementary Figure 17. **Momentum space representation.** Images of cavity modes computed in the XZ plane and at the indicated wavelengths and their Fourier Transformed power spectra for a complete microsphere with R = 0.6 micron (top set), R = 1.22 micron (middle set) and R = 1.8 micron (bottom set) when excited at the side (see Supplementary Figure 4) under the geometry described in Supplementary Figure 1. At the top of each image we provide the value of angular order m of the WG mode which seems to dominate in the image.

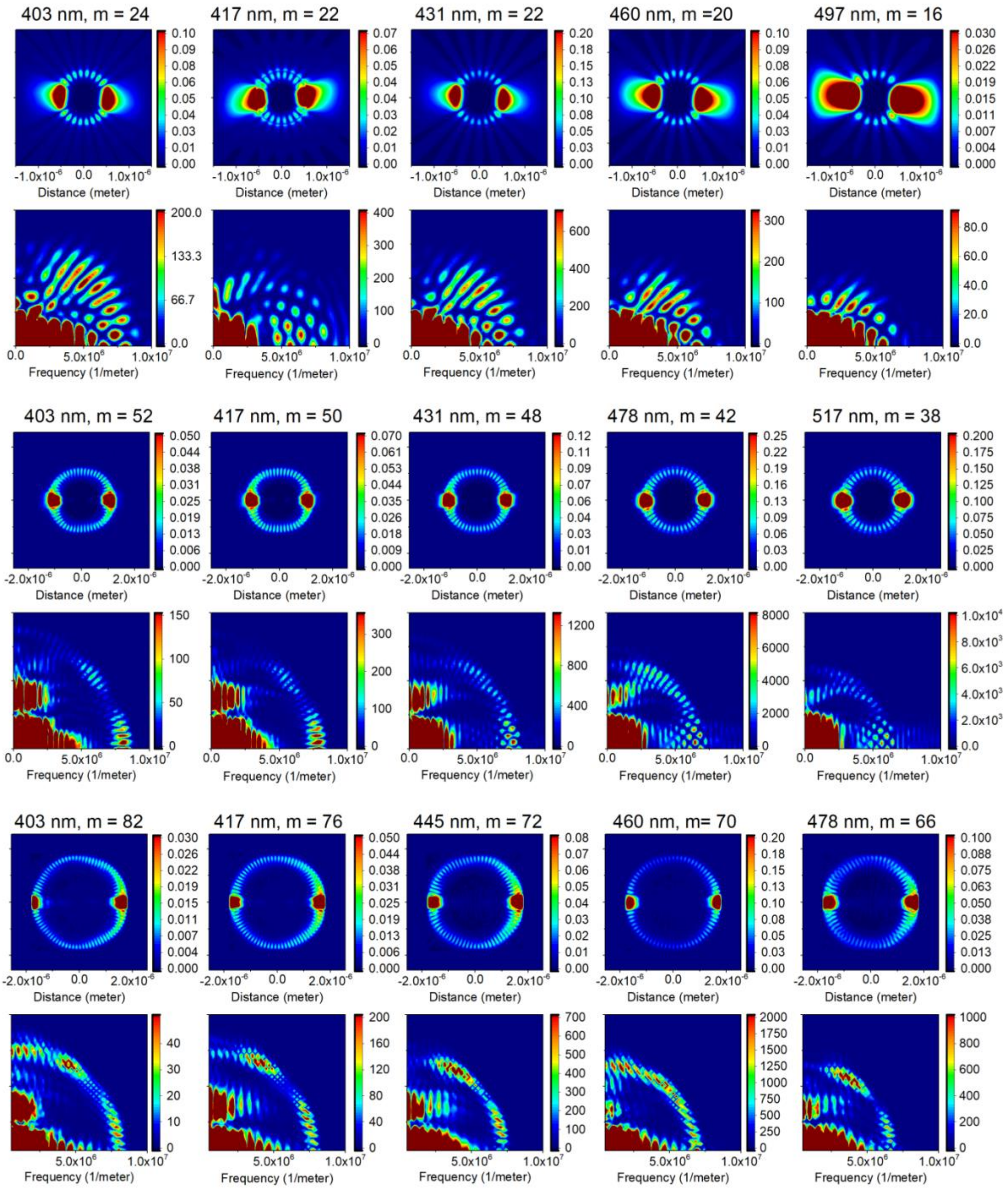

Supplementary Figure 18. **Momentum space representation.** Images of cavity modes computed in the XY plane and at the indicated wavelengths and their Fourier Transforms power spectra for a complete microsphere with R = 0.6 micron (top set), R = 1.22 micron (middle set) and R = 1.8 micron (bottom set) when excited at the side (see Supplementary Figure 4) under the geometry described in Supplementary Figure 1. At the top of each image we provide value of angular order m of the WG mode which seems to dominate in the image.

Supplementary Note 11. RAMAN RESPONSE IN DEPENDENCE ON WAVELENGTH

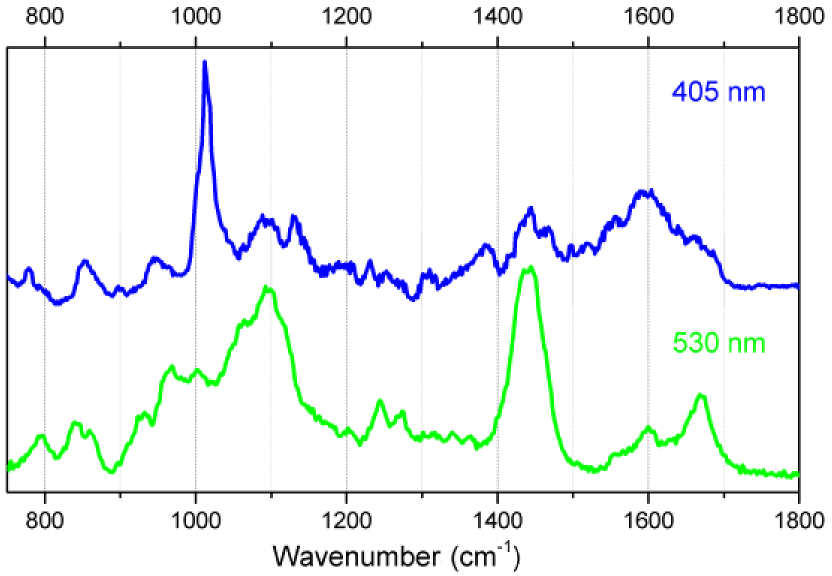

Supplementary Figure 19. **Comparison of Raman spectra of the G12 dodecapeptide when next to ZnO microspheres deposited on glass substrate under different excitation wavelength**. The upper spectrum was taken with a 100X (0.8 NA, 3.4 mm working distance) using excitation at 405 nm. The lower spectrum was taken with a 100X (1.23 NA, 1.8 mm working distance) using excitation at 532 nm.

The spectrum under UV excitation clearly indicates strong relative enhancement of the Amide II contribution. This is due to the resonance with the backbone of the dodecapeptide. Differences in the CH_2 and Amide I regions may be ascribed to enhancement of the interfacial modes stimulate by 405 nm excitation of the field component in ZnO.

SUPPLEMENTARY REFERENCES

1. Moirangthem, R. S. et al. Optical cavity modes of a single crystalline zinc oxide microsphere. *Opt. Expr.* **21,** 3010-3020 (2013).

2. Limo, M. & Perry, C. C. Thermodynamic Study of Interactions between ZnO and ZnO Binding Peptides Using Isothermal Titration Calorimetry. *Langmuir* **31,** 6814-6822 (2015).

3. Stelling, C. et al. Plasmonic nanomeshes: their ambivalent role as transparent electrodes in organic solar cells. *Sci. Rep.* 7, 42530 (2017).

4. Mandelshtam, V. A. & Taylor, H. S. Harmonic inversion of time signals and its applications. *J. Chem. Phys.* **107,** 6756 (1997).

5. Guillet, T. & Brimont, C. Polariton condensates at room temperature. *Comptes Rendus Physique* **17,** 946-956 (2016).

6. Balanis, C. A. *Advanced Engineering Electromagnetics* (Wiley, 1989), p. 566.

7. Boyd, G. D. & Kogelnik, H. Generalized confocal resonator theory. *Bell System Tech. J.* **41,** 1347-1369 (1962).
